# Sociocultural Factors Affecting Breastfeeding Practices of Mothers During Natural Disasters: A Critical Ethnography in Rural Pakistan

**DOI:** 10.1177/23333936221148808

**Published:** 2023-01-25

**Authors:** Shela Akbar Ali Hirani, Solina Richter, Bukola Salami, Helen Vallianatos

**Affiliations:** 1 The University of Regina, Canada; 2 The University of Saskatchewan, Canada; 3 The University of Alberta, Canada

**Keywords:** breastfeeding, disaster, relief camps, sociocultural, facilitators, barriers, mothers, Pakistan, مطلوبہ الفاظ, ماں کا دودھ, قدرتی آفات, امدادی کیمپ, سماجی ثقافتی, سہولت کار, رکاوٹیں, مائیں, پاکستان

## Abstract

Natural disasters affect the health and well-being of mothers with young children. During natural disasters, this population is at risk of discontinuation of their breastfeeding practices. Pakistan is a middle-income country that is susceptible to natural disasters. This study intended to examine sociocultural factors that shape the breastfeeding experiences and practices of internally displaced mothers in Pakistan. This critical ethnographic study was undertaken in disaster-affected villages of Chitral, Pakistan. Data were collected utilizing multiple methods, including in-depth interviews with 18 internally displaced mothers and field observations. Multiple sociocultural factors were identified as either barriers or facilitators to these mothers’ capacities to breastfeed their children. Informal support, formal support, breastfeeding culture, and spiritual practices facilitated displaced mothers to sustain their breastfeeding practices. On the other hand, lack of privacy, cultural beliefs, practices and expectations, covert oppression, and lack of healthcare support served as barriers to the breastfeeding practices of displaced mothers.

## خلاصہ

قدرتی آفات چھوٹے بچوں والی ماؤں کی صحت اور تندرستی کو متاثر کرتی ہیں۔ قدرتی آفات کے دوران، پریشانیوں کی وجہ سے مائیں دودھ پلانا بند کر سکتی ہیں۔ پاکستان ایک درمیانی آمدنی والا ملک ہے جو قدرتی آفات کا شکار ہے۔ اس مطالعے کا مقصد سماجی ثقافتی عوامل کا جائزہ لینا تھا جو پاکستان میں اندرونی طور پر بے گھر ہونے والی ماؤں کے دودھ پلانے کے تجربات اور طریقوں کو تشکیل دیتے ہیں۔ یہ تنقیدی ایتھنوگرافک تحقیق پاکستان کے چترال کے آفات سے متاثرہ دیہاتوں میں کیا گیا تھا۔ متعدد طریقوں کا استعمال کرتے ہوئے ڈیٹا اکٹھا کیا گیا، بشمول 18 اندرونی طور پر بے گھر ہونے والی ماؤں کے ساتھ گہرائی سے انٹرویوز اور فیلڈ مشاہدات۔ متعدد سماجی ثقافتی عوامل کی شناخت ان ماؤں کی اپنے بچوں کو دودھ پلانے کی صلاحیتوں میں رکاوٹوں یا سہولت کار کے طور پر کی گئی۔ غیر رسمی مدد، رسمی مدد، دودھ پلانے کی ثقافت، اور روحانی طریقوں نے بے گھر ماؤں کو اپنے دودھ پلانے کے طریقوں کو برقرار رکھنے میں سہولت فراہم کی۔ دوسری طرف، دودھ پلانے کی جگہ کی کمی، ثقافتی عقائد، طریقےاورتوقعات، ڈھکے چھپے جبر،اور صحت کی سہولیات کا فقدان بے گھر ماؤں کو اپنا دودھ پلانے میں رکاوٹوں کا سبب بنتے ہیں۔

Natural disasters and displacement affect the health and well-being of women with young children (Fothergill & Squier, 2018). During natural disasters, women often face violence, oppression, exploitation, compromised health, neglect, and risk to personal safety (Enarson, 1999; Fatema et al., 2019; Fothergill & Squier, 2018; Hirani & Richter, 2019). Humanitarian aid distributed during natural disasters often lacks gender sensitivity which negatively affects maternal functioning surrounding breastfeeding and child-rearing (Hirani et al., 2021). During natural disasters, women with young children in low-and middle-income countries are often settled in disaster relief camps that lack necessities of life, such as clean water, sanitation facilities, privacy, and adequate shelter (Hirani & Kenner, 2011; MirMohamadalile et al., 2019; Mudiyanselage et al., 2022; Sulaiman et al., 2016). Young children living in disaster relief camps are more prone to malnutrition, compromised immunity, and increased susceptibility to infectious diseases due to suboptimal breastfeeding practices and the use of formula milk prepared in unclean water (Carothers & Gribble, 2014; Hirani, 2021; Hirani et al., 2021; Mudiyanselage et al., 2022). Early cessation of breastfeeding, use of unsafe water, and resultant water-borne diseases are reported to cause the deaths of 760,000 young children each year during natural disasters (World Health Organization [WHO], 2016). Breastfeeding is ideal nutrition that can save the lives of more than 820,000 young children each year, especially during natural disasters (Branca & Schultink, 2016; WHO, 2021).

Pakistan is one of the low-middle-income countries where natural disasters are prevalent and compromised child health is one of the serious repercussions of natural disasters (Hirani, 2014). The geographic location of this country increases its susceptibility to recurrent natural disasters, including hurricanes, heat waves, earthquakes, drought, and flooding (Hirani, 2012). This country has the second-highest infant and child mortality rates in South Asia mainly due to early cessation of breastfeeding and early initiation of supplementary foods that lead to malnutrition, compromised immunity, communicable diseases, and water-borne diseases among children under 5 (Hirani, 2012; Hirani et al., 2019; Ullah et al., 2014; UNICEF, 2013). During disasters, child mortality in Pakistan often increases by approximately 10% because of a further decline in breastfeeding prevalence and subsequent rise in childhood morbidities (Hirani, 2014, 2021; Hirani & Kenner, 2011; Warraich et al., 2011). UNICEF (2018) reports that among Pakistani mothers, only 20% of women initiate breastfeeding within 1 hour of birth, 48% of mothers exclusively breastfeed their infants for 6 months, and 57% of mothers breastfeed their babies until 2 years while providing complementary feeding. Due to limited research on the breastfeeding practices of Pakistani mothers in disaster relief camps, the prevalence of breastfeeding during disasters, and the reasons behind sub-optimal breastfeeding practices of internally displaced mothers are not available. Also, very little research has been undertaken to examine a range of sociocultural factors that affect the breastfeeding practices of mothers in disaster relief camps in Pakistan.

During natural disasters in Pakistan, affected families are resettled in temporary disaster relief camps usually placed far from the city (Hirani, 2014). In these camps, internally displaced women with young children live in cramped situations, are often dependent on donated basic supplies, and experience trauma associated with displacement (Hirani, 2014; Hirani et al., 2019, 2021; Maheen & Hoban, 2017; Sadia et al., 2016). Given the rising child mortality rates during a disaster and the decline in breastfeeding prevalence, a comprehensive understanding of the range of sociocultural factors that affect the breastfeeding practices of internally displaced mothers is crucial, especially for those residing in disaster relief camps after natural disasters. Although the previously undertaken studies with internally displaced women in Pakistan provide insight into the key challenges encountered by women, especially pregnant mothers, these studies do not uncover the sociocultural factors that shape the breastfeeding practices of internally displaced mothers residing in disaster relief camps. Previously undertaken studies highlight a lack of privacy as a major challenge to breastfeeding in the setting of a disaster relief camp and the resultant embarrassment experienced by internally displaced mothers (Bukhari & Rizvi, 2015; Maheen & Hoban, 2017). However, these studies do not investigate the range of sociocultural factors that shapes the breastfeeding practices and experiences of mothers in the setting of a disaster relief camp. Also, a limited number of empirical studies have been undertaken that focus on the facilitators and barriers at the sociocultural level that shape the breastfeeding practices of internally displaced mothers. Hence, there is a need to explore sociocultural factors that positively and negatively affect the breastfeeding practices of internally displaced Pakistani mothers residing in disaster relief camps. This study aimed to examine sociocultural factors that shape the breastfeeding experiences and practices of internally displaced mothers who are affected by natural disasters and residing in disaster relief camps in Pakistan.

## Methods

### Study Design

This study was undertaken using a critical ethnographic design. Critical ethnography provides an opportunity to critically examine issues surrounding the lives of people facing vulnerability, conflict, and struggles (Cook, 2005; Groenkjaer, 2002; Harrowing et al., 2010). This design provides an opportunity to examine the experiences of the oppressed group living in a particular culture and to analyze the association of those experiences within specific power relationships (Harrowing et al., 2010). This design assists in gaining an in-depth understanding of the range of factors in social structures that shape the experiences and practices of the vulnerable and oppressed groups (Cook, 2005), in this case breastfeeding mothers affected by natural disasters and residing in the disaster relief camps of Pakistan. In comparison with other forms of ethnography, critical ethnography presents a unique perspective of involving participants in the iterative process of data collection and data analysis, critically examining their experiences, and facilitating them to reflect on why a problem exists (Cook, 2005; Madison, 2012).

In this study, the critical ethnographic study design provided an opportunity to uncover and critically examine the range of sociocultural factors (facilitators and barriers) that shape the breastfeeding experiences and practices of the vulnerable group of internally displaced mothers residing in the disaster relief camps of Pakistan. In Pakistani society, where internally displaced women are often exposed to gender-based violence, oppression, food insecurity, and health inequalities (Asad et al., 2013; Carballo et al., 2005; Nour, 2011), critical ethnography was viewed as suitable to establish a meaningful dialogue with the marginalized group of internally displaced mothers and learn about sociocultural factors that are directly and indirectly affecting their breastfeeding practices.

### Theoretical Framework

The theoretical framework that guided this study is Amartya Sen’s capability approach. The capability approach framework by Sen (2005) provided a conceptual ground to examine the dynamic association and interrelationship between maternal agency and contextual factors in the sociocultural environment that shape the breastfeeding experiences and practices of displaced mothers affected by natural disasters. This framework identifies five key concepts, including capabilities, functioning, agency, endowment, and conversion factors (Sen, 1999a, 1999b). *Capabilities* refer to “possible and available opportunities to an individual”; *functioning* refers to “choices, values, and willingness to pursue the possible and available opportunities”; *agency* refers to “ability to act”; *endowment* refers to “available resources or support in the environment” (i.e., physical, mental, social, or public) that reinforces the capabilities and functioning of an individual; and *conversion factors* refer to the wide range of factors that affect the capabilities and functioning of an individual (Hirani & Richter, 2017; p. 52). Sen’s capability framework provided an opportunity to first identify the range of contextual factors (sociocultural facilitators and barriers) and then analyze the mechanisms through which these factors affect the agency of displaced mothers pertinent to their breastfeeding practices.

### Setting

This study was undertaken in Chitral, Pakistan where natural disasters are prevalent. Chitral is situated in the Khyber Pakhtunkhwa province of Pakistan with an approximate population of over 479,000 people (Pakistan Poverty Alleviation Fund, 2015). Chitral is a mountainous region located in the extreme north of Pakistan (lies at an average elevation of 1500 meters above sea level). In 2015, thousands of families in Chitral were affected by the Glacial Lake Outburst Flooding and subsequent earthquake. The recurrent disasters in this region resulted in many families living in temporary settlements, where people are housed in tents, transitional shelters allocated by the disaster relief agency, or makeshift huts built out of mud and brick. After receiving ethics approval from the University of Alberta’s Research Ethics Board (No. Pro00070613), data were collected in four different disaster-affected villages of Lower Chitral, including Shali, Bumburate (Kalash valley), Zhitoor (Garam Chashma valley), and Beshqair (Garam Chashma valley). The principal investigator (Shela Hirani) who undertook fieldwork accessed these villages, with the support of a humanitarian relief agency based in Pakistan, by travelling through unstable roads on mountains and using ground transportation to gain access to the participants residing in those villages.

### Sample and Participant Recruitment

Emergent, purposive, and maximum variation sampling methods were utilized to recruit a sample of 18 displaced women who had young children aged 1 day to 36 months. Mothers were eligible to participate in the study regardless of their breastfeeding practices. A local community mobilizer as a member of the humanitarian relief agency supported the participant recruitment by identifying displaced women who met the inclusion criteria and invited them to participate in this study. The community mobilizer was a locally trained woman having proficiency in the local languages, knowledge of safety measures, and thorough awareness of cultural norms and daily routines of women with young children.

Internally displaced mothers who accepted the invitation and met inclusion criteria were then contacted by the principal investigator (Shela Hirani). The participants were told that their participation was completely voluntary and that they would have the option to withdraw their data by contacting the researcher during the fieldwork of this study. Participants were assured of their confidentiality during the process of data collection, analysis, reporting, and dissemination.

### Data Collection

Data were collected by the principal investigator (Shela Hirani). Multiple methods were utilized to collect data, including field observations, document reviews, and in-depth interviews with internally displaced women. Use of multiple methods assisted in triangulating the data and gaining an in-depth understanding of the phenomenon. The field observations were selective and specific to internally displaced mothers and available breastfeeding support for these mothers in their sociocultural context. The principal investigator (Shela Hirani) observed the environmental resources/facilities available for mothers (clothes, space, specific feeding supplies, food portion provided to women, and privacy issues for breastfeeding mothers) at the identified disaster relief setup; roles and responsibilities that mothers were undertaking during the day (division of labor, leisure time, etc.); and attitudes of people (family members, healthcare professionals, relief workers, and volunteers) toward mothers (supportive or non-supportive). During fieldwork, the principal investigator (Shela Hirani) also gathered information from the documents, such as flyers, newsletters, and reports maintained by the relief organization and health units in Chitral to gain insight into the support services offered to the disaster-affected community and breastfeeding mothers residing in relief camps.

In-depth interviews were conducted in the national language of Pakistan (Urdu) and were audio recorded. Before conducting in-depth interviews, the principal investigator (Shela Hirani) ensured that the identified location was private, comfortable for the participants, and free of distractions. As per participants’ preference, interviews were conducted in the shelters while their infants were sleeping or breastfeeding. More than one interview was undertaken with four participants to assure the sufficiency of data, triangulate data gathered through field observations, and seek clarification on specific aspects of the facilitators and barriers toward the breastfeeding practices of mothers. The audio-recorded interviews in Urdu were translated into English and then transcribed by a transcriptionist who had proficiency both in Urdu and English. The principal investigator (Shela Hirani) undertook the audit trail of all the interviews to verify the translation and content of the interview. Ten percent of the translated verbatim in English were translated back into Urdu by a language expert to check the accuracy of the translated version.

The principal investigator (Shela Hirani) adopted a variety of strategies to assure the rigor and trustworthiness of data during and after the fieldwork in the disaster relief camps of Chitral, Pakistan. The strategies included a demonstration of a reflective approach (self-reflexivity), critical consciousness, practical wisdom, cultural immersion, mindfulness of positionality, triangulation and trust-building strategies with the participants, and maintenance of prolonged engagement with the relief organization. Moreover, the principal investigator’s positionality of being a partial insider and partial outsider facilitated in embracing of new knowledge, perspectives, and diverse aspects of the culture during the data collection. The principal investigator (Shela Hirani) was an insider to this research because she is a Pakistani female with proficiency in the national language (Urdu), a mother with prior experience in breastfeeding, as well as a nurse and lactation consultant with prior experience of conducting research in low-income and semi-urban settings in Pakistan, particularly with vulnerable women. However, simultaneously the principal investigator (Shela Hirani) approached this research as an outsider because she was born and raised in an urban city in Pakistan. She is an educated Muslim female who belongs to an upper-middle class family, who was pursuing her PhD in nursing, and who had been living in Canada for 2 years prior to data collection. She had no prior personal experience of being internally displaced and managing breastfeeding while residing in a disaster relief camp.

During fieldwork, the principal investigator (Shela Hirani) adopted the customs (ways of meeting and greeting), followed the community’s way of dressing, established rapport with the health care team, community leaders and displaced families in the setting of disaster relief camps, and engaged in a non-judgmental manner with participants during the data collection, especially during in-depth interviews with participants. During in-depth interviews, the principal investigator (Shela Hirani) engaged in meaningful dialogues and discussions with participants, allowed them to talk more, ask questions, or seek clarification. The built rapport and trusting relationships with the participants facilitated them to talk and discuss challenges associated with their breastfeeding practices without any hesitation or shyness.

### Data Analysis

Data analysis was inductive and iterative. Data gathered through in-depth interviews, field observations, and review of reports/newsletters were initially analyzed manually by the principal investigator (Shela Hirani). Using a critical lens, several steps were followed during data analysis. The initial step involved the selection and isolation of codes revealing socio-cultural factors that are directly and indirectly affecting breastfeeding practices during natural disasters. The next step involved a comparison of information gathered through multiple sources, including in-depth interviews, document analysis, and field observations. This helped in the validation of the data and identification of patterns in the data set. The next step involved comparison, contrast, and identification of categories surrounding the notion of sociocultural factors that facilitate or impede breastfeeding practices of internally displaced mothers in disaster relief camps. Color coding was used to categorize whether the derived information (codes) are sociocultural facilitators or barriers. As a final step, all the emerging patterns within the data set (in-depth interviews, document analysis, and observation) were further analyzed and compared to identify broad themes. During the fieldwork, the principal investigator contacted the participants (in person or via phone) to verify the interpretations drawn from the data. To assure the trustworthiness of the data and rigor in the process of data analysis, one of the co-investigators (Solina Richter) audited the data gathered from multiple sources, undertook independent coding of the data gathered through multiple sources, and actively participated in the process of derivation of categories and themes. A codebook was developed having a list of the codes, categories, and themes derived from the findings gathered through multiple methods. While reporting the study findings, participants’ anonymity was assured by using identification numbers instead of their real names.

## Findings

### Description of Participants

The participants’ ages ranged from 18 to 40 years. The education level of the mothers varied from illiterate to university education (Bachelor of Science). Their religious backgrounds included Sunni Muslim, Ismaili Muslim, and Kalashi. The participants were from diverse ethnic backgrounds including Bazaki, Jalendari, Kalash, Katoray, Musingay, Gajani, Darwaish, Jhatak, Turkali & Dhundaray. The mother tongue of the majority of the participants was Khowar, whereas the first language of a few participants included Pushto, Ludhvi, Kalashi, and Nuristani. The majority of the participants were living in an extended family system (with in-laws, grandparents, husband, and children), whereas 4 out of the 18 participants were living in a nuclear family system (with husband and children). The total number of people in their household ranged from 4 to 15 members. The total number of children per participant ranged from one child to seven children. The age of the youngest children ranged from 3 months to 3 years. The sex of most of the youngest children was male (altogether 10 boys and 8 girls). At the time of interviews, 3 of the 18 mothers were exclusively breastfeeding their infants, 11 were feeding breastmilk along with cow’s milk, solids, and/or formula milk, and 4 mothers were using breast-milk substitutes (cow’s milk, formula milk, and/or solids) to meet the nutritional requirement of their youngest child. Ongoing natural disasters, including floods, earthquakes, and landslides had made rebuilding houses extremely challenging, and consequently, the participants had been forced to reside in a variety of disaster relief temporary housings (shelter, mud brick house, and tents) for as long as 2.5 years. Demographic characteristics are summarized in Table 1.

**Table 1.** Summary of the Demographic Characteristics of the Study Participants.

Characteristics	Findings (*n* = 18)
Age range	18–40 years
Villages	
Bumburate	3
Beshqair	4
Shali	1
Zhitoor	10
Education	
Illiterate	8
Grade 1–10	6
Bachelor’s degree	4
Mother tongue	
Khowar	13
Others	5
Religion	
Sunni	4
Ismaili	13
Kalashi	1
Type of family	
Nuclear	4
Extended	14
Type of housing	
Angle iron shelter	14
Tent	3
With relative in a kutcha house	1
Age of youngest child	
Less than 1 year (0–11 months)	5
1–2 years (12–24 months)	10
3 years (25–36 months)	3
Type of feeding	
Exclusive breastfeeding	3
Breastmilk along with breast-milk substitute	11
Breast-milk substitute (cow’s milk, formula milk, and/or solid food)	4

### Sociocultural Factors as Facilitators and Barriers to Breastfeeding Practices

**Figure 1.**
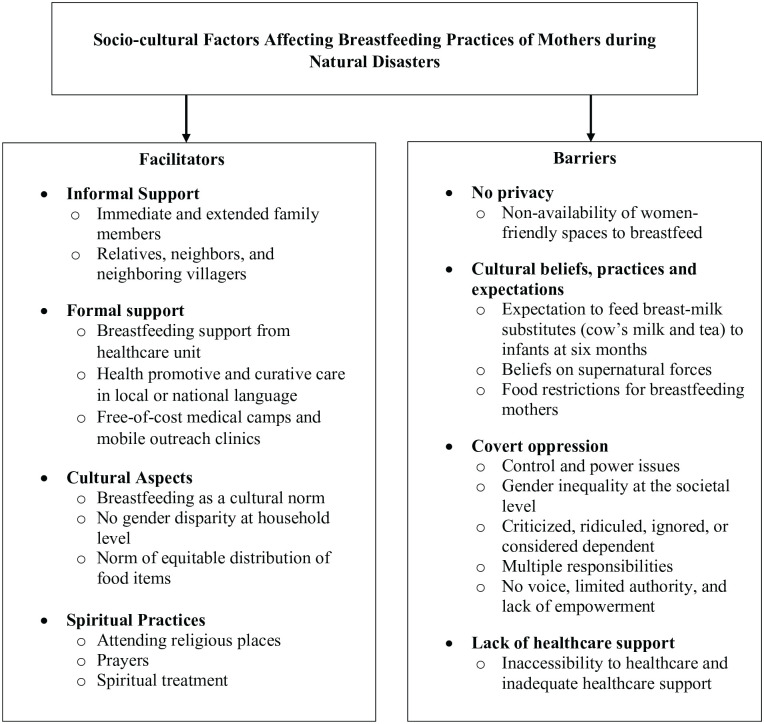
Socio-cultural factors affecting breastfeeding practices of mothers during natural disasters.

Figure 1 describes sociocultural factors that were serving as facilitators and barriers to breastfeeding practices of internally displaced mothers during natural disasters in Pakistan. The derived themes and categories from data gathered through in-depth interviews, documents, and field observations are discussed below.

### Facilitators Affecting Maternal Capabilities and Functioning

Sociocultural factors that serve as facilitators toward breastfeeding practices of mothers residing in disaster relief camps include informal support, formal support, culture, and spiritual practices. Each of these categories is discussed below:

#### Informal support

Participants acknowledged the informal support toward their breastfeeding practices from their immediate and extended family members (parents, husband, in-laws, and older children), as well as from their neighbors, and neighboring villagers, including both females and males. The type of support included nutritional guidance, provision of a special diet (milk and goat’s meat broth) to increase breastmilk supply, encouragement for breastfeeding, financial help, accommodation at a relative’s place at the time of the disaster, donated supplies to meet daily necessitates (food, milk, and clothes), assistance in meeting child-care responsibilities and household chores, and land for placing the temporary shelter or tent. A study participant, who was facing ongoing, recurrent earthquakes of low intensity in her village and had a 3-month-old child who was born during her settlement in the temporary housing, acknowledged the *support received from the mother-in-law and the immediate family members* concerning breastfeeding and the importance of maintaining adequate nutrition:

My mother-in-law told me that if I pay proper attention to my diet then he [child] will be fine; otherwise, if I don’t pay attention to the diet then he [child] will not get the proper amount of breastmilk which he should be getting. . . . My family had a goat slaughtered and gave me the broth for strength. They also gave me cow’s milk in the morning so that my diet would be nutritious and that I could be healthy; in return, the child would also get properly nursed.

Another woman who was living in a transitional shelter and continuing her breastfeeding practices acknowledged a similar kind of support. She verbalized:

My mother would tell and guide me when I wasn’t able to breastfeed the child; she told me this is how I should breastfeed the child. . . . She told me how to lay him [child] down properly and breastfeed him. . . . They gave me fresh meat broth. They sacrificed the goat in the house and gave me its broth.

Field observations supported what mothers described, particularly with support in cooking, cleaning, child care, care of cattle, and fieldwork from their family members and neighbors. Participants believed that this informal support enabled them to cope and look after their child-care responsibilities during a challenging time. Participants also acknowledged the support received from their relatives and neighboring villagers who provided them refuge during the initial few days of the disaster, arranged food and clothes for them, and offered them land on which to place a shelter or tent.

#### Formal support

While informal supports were critical for survival post-disaster, formal support from governmental and non-governmental agencies was also important in the immediate aftermath of a disaster. Participants particularly acknowledged the *formal support from the health care units*. Most of the participants mentioned that the nutritional guidance from the health units during the period of disaster and displacement improved their health and facilitated the re-establishment of their breastmilk supply during their settlement in disaster relief camps. For example, a participant, who experienced low breastmilk supply soon after the disaster and fed cow’s milk as a temporary breastmilk substitute to her infant, described the role of the disaster health unit in re-establishing breastfeeding. She shared, “I went to the doctor and got myself checked. They gave me medicines and advised me to focus more on my diet, and gradually my milk supply came back to normal.”

A few other participants acknowledged the curative and health promotive aspect of the care that they received in their local or national language, including medical treatment of illness, prenatal guidance about breastfeeding, measures to prevent malaria and flu, health checkups and visits, health and hygiene training, and informational booklets in Urdu (the national language of Pakistan). The information gathered from the documents, mostly newsletters and reports maintained by the health unit also supports that at the time of the disaster several awareness sessions, health camps, and vaccination campaigns were organized for the disaster-affected communities. These services were offered by locally-trained community workers and healthcare providers, including female health visitors, vaccinators, doctors, and nurses. As disaster-affected communities could not access the healthcare setting due to health issues, non-availability of transportation, and damaged roads after a major disaster, the locally-trained healthcare providers and volunteers (nurses and doctors) from the southern parts of Pakistan formed a mobile team and travelled to the disaster-affected areas, set up free-of-cost medical camps and mobile outreach clinics, and offered health promotive, medical, and emergency services to many disaster-affected families.

Although health units were not accessible to most of the displaced families because of the scattered nature of the temporary housing across the landscape, there were a few participants whose shelters/tents were located near the health unit, and this facilitated their access to formal support related to breastfeeding. This does suggest that for mothers without this kind of access to health support, continuing to breastfeed in the months post-disaster may become increasingly challenging.

#### Cultural aspects

Participants shared various positive aspects of their culture that help them to sustain their breastfeeding practices even during the stressful time of the disaster, displacement, and relocation in the disaster relief camps. Participants shared that despite the barriers imposed by disaster and displacement, breastfeeding as a cultural norm encourages them to sustain their breastfeeding practices. Most of the participants shared that they exclusively breastfeed until 6 months and then continue to breastfeed until up to the age of 3 to 5 years while supplementing breastmilk with cow’s milk and solid foods. Participants further shared that as soon the child is born, female members in their family or neighborhood usually encourage them to breastfeed their first milk (colostrum) to the newborn and provide support in positioning the newborn during latching. During in-depth interviews, participants further shared that they only opt for feeding formula milk when a mother dies, is too sick, or is unable to produce adequate breast milk. There was no evidence of wet nursing among participants. The only available feeding options for the young children included breastmilk, cow’s milk (mainly cow and goat’s milk), solid foods, or formula milk if nothing is available. A participant who continued breastfeeding her child during and after the disaster acknowledged the *norm of breastfeeding in the village* that facilitates breastfeeding practices of nursing mothers. The participant shared:

In our village, all the mothers breastfeed their children. They don’t use the milk [formula milk] from the store because we live in a village and people don’t have jobs here or studies, so that is why they nurse their children themselves and they don’t require store-bought milk.

Participants acknowledged that at the household level there was *no discrimination* when it comes to breastfeeding daughters and sons. Participants’ stories regarding emergency evacuation revealed that they assured the safety of all their children (girl or boy), and despite prolonged hunger, they continued to breastfeed their children regardless of their sex. Participants also acknowledged that at the household level there is a norm of *equitable distribution of food items and humanitarian aid*. A participant, who believed that this household norm facilitated her to sustain her breastfeeding practices while living in the temporary housing (tent and transitional shelter), shared:

I did get the support [food items as humanitarian aid] but it wasn’t the same as what we had before in our homes. We were surviving in those times. It [food] was equally distributed among all the family members, no one used to get less or more food.

A few other participants believed that this norm of equitable distribution of food items provides nursing mothers with an equal opportunity to look after their nutritional needs while experiencing the stress of disaster, displacement, and settlement in the relief camps. These participants further acknowledged that depending on the availability of food, displaced women often decide to eat less and give more food to their children, elderly kin, and male members who work outside the home in fields. This indicated that in view of the economic circumstances and availability of food, women often go against this household-based cultural norm in view of their personal choice and societal values that expect women to sacrifice whenever required.

#### Spiritual practices

During fieldwork, it was noticed that attending a religious place like a mosque, shrine, or prayer hall is a common practice among families belonging to all religions. Although there were few or no health care settings in the villages where fieldwork was undertaken, most of the villages had spiritual healing clinics meant to offer spiritual advice, as well as amulets and exorcize possessions in order to alleviate sickness and improve well-being. There was a cost involved in seeking spiritual treatment; one of the participants shared that the cost is typically Pakistani Rupees 10,000/- to 25,000/- (USD 45–150). As these clinics were more accessible, many people in villages who could afford the cost of spiritual treatment opted for healing in these clinics.

Participants who could not sustain their breastfeeding practices after the disaster and did not find medical treatment effective enough shared that they would prefer to receive spiritual support or treatment if they had the money. Participants also acknowledged that *prayers* offered by relatives to enhance the well-being of nursing mothers and *spiritual treatments* administered by spiritual healers both affect mothers’ well-being and their abilities to fulfill their maternal responsibilities. A participant who was unable to sustain her breastfeeding practices right from the time of disaster underscored that an arrangement of *prayers* by her community people or relatives could have facilitated her to re-establish her breastfeeding practices. She verbalized:

Based on my belief, I think that when my milk got dried up at that time [during a disaster]. If someone would have given me blessings and arranged prayers for me then that would have been helpful to re-establish my milk supply instantly. But no one did that for me.

Another participant who reported ill health due to the influence of possessions by supernatural forces (*Saya* is the local term), and who was unable to sustain her breastfeeding practices, considered that *spiritual healing often* supported her recovery from the influence of these possessions and facilitated her ability to resume her child-care responsibilities.

### Barriers Affecting Maternal Capabilities and Functioning

Sociocultural factors that served as barriers to the breastfeeding practices of mothers residing in the disaster relief camps include no privacy, cultural beliefs, practices and expectations, covert oppression, and lack of healthcare support. Each of these categories is discussed below:

#### No privacy

Lack of privacy to breastfeed was identified as the top barrier affecting the breastfeeding practices of mothers affected by natural disasters. All the participants shared that during a disaster, displacement, and their settlement in the relief camp (shelter or tent), there was no privacy or availability of women-friendly spaces to breastfeed. Participants were of the view that their privacy continued to be jeopardized while living in overcrowded tents and shelters. They felt uncomfortable to rest and breastfeed their young children in front of their relatives, male family members, and guests. A participant who was living in a tent and substituting breastmilk with cow’s milk shared:

I faced lots of troubles when my child was born. It was cold, and everyone [family members] was there. I couldn’t sleep comfortably or get relaxed, so faced lots of breastfeeding issues in this [tent]. I had trouble when people would come in [guests and relatives] while I was breastfeeding the child. I could not sleep at all. I couldn’t breastfeed my child in front of them [guests and relatives].

A participant, who on encountering the challenge of lack of privacy to breastfeed made sure to cover herself with her long shirt and scarf while breastfeeding the child but could not sustain her breastfeeding practices, shared, “There is a single room [shelter] and all the people in the family share this space. It gets difficult when the child is screaming and removing the long scarf (dupatta in local term) from over her [child] during breastfeeding.”

Women in Chitral often restrict their movement to certain locations in the village, such as canals, fields, prayer halls, and a neighbor’s house. The participants mentioned that during disaster and displacement when they had to run too far places to find refuge, they had nothing to cover themselves, had no privacy to breastfeed, and felt “unfenced” (in the state of *Bay pardagi* in Urdu). While sharing the nature of the painful experience, one of the participants said:

When the floods came in, we were left completely unfenced and became unveiled (“*bay pardha*”). We didn’t have shelter at that time, we lived in a tent for a month. The men would live outside the tent, whereas the women lived inside the tent. The mothers and children were also living inside the tent. There were no clothes for the children. They only had what they were wearing. Whenever I had to breastfeed my child, there was no privacy (“*bay pardagi*”). We couldn’t breastfeed them [children] comfortably like a mother does in her own house. There were difficult times.

While sharing the challenge of breastfeeding in open spaces with no privacy, another participant who initially had no place to live stated:

When you are homeless as a mother it becomes very difficult. It becomes embarrassing when you have to breastfeed your child and you are on the streets and in an open space. It doesn’t feel good nor is it something that a mother feels comfortable breastfeeding. It was very painful for us as there were many people on the streets who passed by and all that time you think that this person is watching you and you keep on adjusting yourself. It wasn’t easy to breastfeed your child at that time.

#### Cultural beliefs, practices, and expectations

Participants shared their cultural beliefs and practices concerning encouragement to feed breast-milk substitutes (cow’s milk and tea) to infants at 6 months, beliefs on supernatural forces, and food restrictions for breastfeeding mothers. Mothers shared that most of the time these beliefs and practices negatively affect their health, breastfeeding practices, and management of their child-care responsibilities, especially during natural disasters and displacement when they try to cope with additional responsibilities, transitions, and stressors. Most of the participants shared that according to their culture and the availability of cattle (mainly cows and goats) in their household they prefer feeding cow’s milk (boiled and diluted) to their children whenever their breastmilk is insufficient. Field observations supported that many families or their acquaintances (relatives and neighbors) had their cow and that cow’s milk was serving as the top choice of breast milk substitute. A participant shared the norm to feed cow’s milk to children when they are 6 months old. She shared:

We breastfeed our children for six months. After six months, we give him [child] some extra food to start with. We give him some cow’s milk and also feed him our milk. When he is old enough and around seven to eight months, then we start by giving him [child] some extra food, such as bread etc.

Tea (*chai*) is one of the most popular cultural drinks, enjoyed by Chitrali families several times a day. Participants shared that they also feed tea to their children from a very early age. One of the participants shared:

We give tea (chai) to children who are young too. My niece who is 19 months old drinks tea; she drinks tea with me in the morning. Because if we drink it alone then they scream and ask for it; so, we start by giving them small sips, and then they grow up to be habitual of that.

A few of the participants shared that in their culture they are expected to feed breastmilk substitutes (mainly cow’s milk) to their infants, especially whenever the child cries due to hunger, the mother’s breastmilk is insufficient, or when the mother is busy with household chores. The field observations supported that each village had a few local stores that were selling household items and formula milk (mainly *Nestlé* products). A participant, who was living in a tent and was supplementing her breastfeeding with cow’s milk, shared that her mother encourages her to initiate breast-milk substitutes (cow’s milk) whenever her child cries in hunger. She shared:

My breastmilk is insufficient so my child cries and screams. My mother scolded me and said that he [child] is hungry, that is why I should give him something extra [other than breastmilk] because now he is seven months old. That is why I feed him cow’s milk and he doesn’t even cry when he is full.

Another participant, who in view of her mother-in-law’s advice was substituting breastmilk with cow’s milk for her 6-month-old child, shared:

I feed her [my child] timely, and sometimes when she [child] cries my mother-in-law gives her cow’s milk. She [mother-in-law] uses a feeding bottle. I add a little water to the cow’s milk because the doctor says that it affects the child’s stomach. So, I add some water and boil it, cool it down, and then pour that into a bottle so that the child drinks it.

A few of the participants mentioned the cultural beliefs of their community people surrounding the *influence of supernatural forces and possessions after the disaster*. They were of the view that after a disaster these supernatural forces have negatively affected women’s health and their child-care responsibilities, including breastfeeding. A participant who could not sustain her breastfeeding practices due to the influence of witchcraft on her health shared:

There are many mothers who believe in the supernatural and possessions (*Saya*). Sometimes they go absolutely quiet and sit in the corner and cry, they don’t take care of their children after that. We strongly believe in those possessions. They [people in the community] also believe that the Jinns and Fairies [supernatural forces] can take over you.

Participants also shared their cultural beliefs and practices surrounding *food restrictions*. A participant talked about the food restrictions for breastfeeding mothers based on cultural beliefs that they have been following as per the received advice from their ancestors and elderly people in the family/community:

They [elderly] say that I [breastfeeding mother] shouldn’t eat cheese because that will be dangerous for the child as he will vomit the food out. They say that if the mothers eat cheese, then the child can vomit the food out. They also avoid sweet food or something sugary because they say that it can cause stomach aches. They don’t allow cow meat saying that it can also be dangerous for the child. Some people follow these regulations while others don’t. There are certain foods which are considered warm but benefit both mothers and children.

#### Covert oppression

In-depth interviews with the participants reflect that at the societal level women often experience covert oppression, specifically during the period of disaster and displacement. Covert oppression refers to hidden forms of control and power issues at the societal level that negatively affect the physical, mental, emotional, and spiritual well-being of women. In Chitral, where women are expected to cover themselves with a long scarf all the time, restrict their mobility to their house or neighborhood, and get the permission of the men/head of the family before going outside, study participants mentioned the challenges and struggles experienced during the period of disaster and displacement. Participants shared that during disasters many women faced criticism as during the emergency evacuation they had to leave the premises of their homes without seeking permission, had no scarf (*dupatta* in local terminology) to cover themselves during an emergency evacuation, and had to live and breastfeed their crying children in open spaces (fields and mountains) having limited privacy. Participants shared that the disaster (glacial lake outburst flooding and flash flooding) continued for many days, hence the relief agency could not reach all of them during the initial few days of the disaster and provide them with food or clothes to cover themselves. As disaster-affected families were escaping from one hill to the other and from one village to the other for several days, participants shared that during this period they had no long scarfs or veils to fully cover themselves, hence they felt embarrassed for going against the cultural norm and were afraid of being judged by relatives and neighboring villagers who were unaffected by the disaster and displacement.

A participant who sustained her breastfeeding practices while juggling multiple responsibilities shared societal expectations for women concerning “birth spacing,” “breastfeeding,” “workload,” and “responsibilities.” Although this participant was 40 years old, she appeared very tired and older than her age. Her quote revealed a few of the key aspects surrounding gender social locations and the gender responsibilities of being a woman:

We do all the work and duties of a man too. Secondly, we feed our children for three years with our milk [breastmilk]. After three years when they [women] stop breastfeeding the child that is when we prepare ourselves for conceiving another one. We do all the hard work and that is why we appear much older than we are. You will find many people like this who will appear as if they are their husband’s mothers but in reality, they are their wives. Men look younger than the women here because we [women] work more than them [men]. This is true for the entire village.

One of the participants who was pregnant shared that for easy labor pregnant mothers are encouraged and are expected to work more (household responsibilities, fieldwork, and cattle care) during the last trimester of pregnancy. The participant was of the view that this societal expectation increases their tiredness to a great extent during the postnatal period, hence indirectly affecting their breastfeeding practices. She shared:

In our culture, it is said that the more a pregnant woman works the sooner her child is born. They say that pregnant mothers should work as much as they can so that the child will be born early in the ninth month. Sometimes the work is too much. I think there are 85 per cent here who work this much; there are a lot fewer people here who don’t force women to work more.

A few of the participants mentioned that after the natural disaster and related economic hardships it is quite common for women to starve themselves or skip meals to fulfill the nutritional needs of their family members and older children. Participants were of the view that this practice negatively affects breastfeeding mothers’ health, nutritional status, and breast milk supply. Although participants acknowledged that at the household level there is no gender inequality, there was evidence of gender inequality at the societal level that was shaping women’s attitudes, behavior, and practices. At the societal level, it was believed that “*being a woman*” means often sacrificing and eating less or skipping meals when there is a shortage of food. The participant stated:

Sometimes you have to take these steps [skip meals] because men work outside, and you have to give the food to your children too. If you aren’t getting some then you need to be silent sometimes. . .women cook food but don’t eat it themselves. They [women] provide for others but skip their meals. You need to manage it for your children and in-laws too.

Another participant shared a norm related to food distribution, “We gave it [food] to those members [family members] who work outside and then to the children; I eat last. Sometimes I get more for myself, sometimes less.”

Participants further shared that disaster and displacement have increased their suffering and have increased their dependency on those who are not affected by disaster or are responsible for aid distribution. They shared that as a primary caregiver when they reach out to people to seek support, they often are ridiculed, ignored, or considered dependent. A participant who did not receive humanitarian aid or a place to live (shelter or tent) could not sustain her breastfeeding practices. She shared her suffering and the reaction of others:

In those times if they [relief agency or donors] had given me a house or taken care of my expenses, then I wouldn’t have been ridiculed amongst the people. The stress which I faced when I had to go to my relatives’ place and ask for help was also very tough for me as that also took a toll on my health. Some of my relatives make fun of me.

The same participant also verbalized her suffering and mentioned that she has no voice, limited authority, and lack of empowerment as being a woman she could not go to the males in the community (responsible for the aid distribution) to ask for humanitarian aid or support needed. She shared:

People [disaster affected] who cannot talk or stand up for themselves aren’t sent forward [to receive aid or support]. I didn’t get anything [humanitarian aid], and neither could I talk to anyone or go in front of a man and demand this. People who are cunning and can speak for themselves are sent forward [to receive aid or support], whereas the others do not get anything. I haven’t gone to any man and complained against this. I did complain to other women, but they couldn’t do anything either.

#### Lack of healthcare support

Although a few of the participants acknowledged the support they received from a healthcare unit, most of the participants considered *inaccessibility to healthcare and inadequate healthcare support* as the major barriers to sustaining their breastfeeding practices during the disaster. A participant who initiated formula feeding during disaster and displacement due to inadequate breastmilk supply shared:

No one [health care team] came here and neither did anyone advise us [about breastfeeding]. I went about with what we thought was right at that time. If they [health workers] would have given me a medicine which I could consume, and my breastmilk flow would increase, I would have readily agreed to that.

Another mother, who could not sustain her breastfeeding practices soon after the disaster and initiated formula feeding, shared, “No one [health care team] came on this site to tell us what to eat and what medicines to take to re-establish the breastmilk supply.”

Another participant talked about the infrequent visits by the health team in the village:

They [health team members] did come here and gave us free medicines once or twice. Ever since the floods, they came in once or twice. They come here almost after every six months so it’s not that helpful for us. We don’t get any medicines when we are sick. (Mother smiled and sighed).

A participant, whose child had dysentery (with fever and stomach ache) and required treatment at a government hospital located 3 to 4 hours away from her village, mentioned, “They [health team] didn’t tell me anything about breastfeeding. They gave us medications and briefed us on the timings when we should use them.”

### Discussion

In this critical ethnographic study, the influence of sociocultural factors was evident in breastfeeding functioning, decisions, behaviors, and practices of displaced mothers residing in disaster relief camps. Through the iterative process of data collection and analysis, we assured that the intersubjective data gathered is reflective of the emic perspective, that is, displaced mothers’ values, beliefs, practices, and attitudes.

The findings suggested that both positive and negative aspects of culture were evident in the lives of displaced mothers who wished to sustain their breastfeeding practices. The presence of support from the formal and informal social networks (both men and women) served as capabilities for the displaced mothers and facilitated them to sustain their breastfeeding practices. In this study, displaced mothers were highly reliant on the social support offered by their immediate family members, health care providers, relatives, and neighboring villagers. Literature also acknowledges that the biggest sources of emotional, tangible, and informational support for breastfeeding mothers are their formal and informal social networks (Gill, 2001; Hong et al., 2003; Raj & Plichta, 1998; Riordan & Gill-Hopple, 2001). Findings reflected that non-availability of support from people in the social network and limited avenues to avail social support were minimizing capabilities and affecting the maternal agency, independence, and control in breastfeeding (functioning).

In the cultural context of Chitral, where women are expected to take care of various responsibilities in and outside their homes and face covert oppression, support from social networks as a key capability was strengthening maternal agency to manage household chores and childcare responsibilities. In this study, the maintenance of social ties with informal social networks was serving as a buffer for displaced mothers during the stressful time of disaster, displacement, and settlement in the relief camps. First-time mothers and young mothers who had limited knowledge about breastfeeding during the natural disaster were not only seeking breastfeeding advice from experienced women in their immediate social network but were also reliant on support from them to fulfill their gender roles and responsibilities. Previous studies that were undertaken with breastfeeding mothers in the context of natural disasters also suggest that social support in the form of constant advice from informal social networks is vital to boost maternal confidence surrounding infant feeding practices during natural disasters (MirMohamadalile et al., 2019; Mudiyanselage et al., 2022).

During the period of disaster and displacement, disruption in the social support network is reported to affect displaced mothers’ functioning, capabilities, agency, autonomy, and independence surrounding breastfeeding (Gribble et al., 2011; Hirani & Olson, 2016; MirMohamadalile et al., 2019; Mudiyanselage et al., 2022). In this study, displaced mothers who lacked social support reported insufficient knowledge about breastfeeding, challenging transition, compromised nutritional status, and difficulties in sustaining their breastfeeding practices. The findings of this study reflected that separation from family members as a result of an emergency evacuation during a natural disaster resulted in the non-availability of support from people in the social network. Also, non-availability or inaccessibility to healthcare professionals after the disruption of the health system constrained mothers’ capacity to overcome challenges surrounding their breastfeeding practices, mainly insufficient breast milk supply. The findings of this study reflected that availability of avenues to avail support from formal social networks, especially breastfeeding counselling, could have had positive effects on displaced mothers’ health and functioning, as well as physical, mental, emotional, spiritual, and social well-being. Evidence from the literature suggests the importance of formal support for breastfeeding mothers. Formal support in the form of one-on-one breastfeeding counselling is reported to promote, protect, and support the breastfeeding practices of mothers (Bhandari et al., 2005; Bica & Giugliani, 2014; de Oliveira et al., 2014; Ochola et al., 2013). Formal support facilitates breastfeeding mothers to share concerns surrounding their infant feeding and seek practical assistance for encountered breastfeeding issues (Lawrence & Lawrence, 2010; Lauwers & Swisher, 2015; MirMohamadalile et al., 2019). It further empowers breastfeeding mothers by enhancing their confidence level, knowledge, self-efficacy, decision-making, and breastfeeding management skills (Lauwers & Swisher, 2015; MirMohamadalile et al., 2019; Mudiyanselage et al., 2022).

The “setting” determines the extent to which mothers can demonstrate their agency and autonomy in breastfeeding (Bloom et al., 2001; Hausman, 2004). Findings suggested that an absence of privacy is a major barrier to valuable functioning and increases the vulnerability of displaced mothers. Pakistan is a patriarchal society where women are required to cover their bodies using long scarves as a religious obligation and cultural norm. In this country, anything concerning breast or breastfeeding is considered a private matter, therefore, not many women openly talk about breastfeeding or prefer breastfeeding in public places (Bukhari & Rizvi, 2015; Hirani et al., 2019; Maheen & Hoban, 2017). Mothers who had no privacy to breastfeed not only experienced stress and psychological aftermaths of disaster but also experienced a negative impact on their breastmilk supply. The findings and recommendations from participants suggested the importance of breastfeeding-friendly housing (in disaster relief camps) and avenues for the displaced mothers that are gender-sensitive as capabilities in the external environment that can promote maternal functioning surrounding breastfeeding. The findings also suggested that the availability of capabilities such as privacy and a safe space for breastfeeding mothers can promote their independence to sustain their breastfeeding practices and goals in the context of disaster relief. Previously undertaken studies with displaced Pakistani mothers in flood-affected districts (Bukhari & Rizvi, 2015) and Sindh province (Maheen & Hoban, 2017) also reported that lack of adequate privacy for women in shelters and disaster relief camps had negatively affected their health, well-being, and safety. The literature further highlights the importance of privacy and safe spaces during disasters to facilitate mothers to make autonomous decisions regarding their breastfeeding practices and improve their functioning (Ayoya et al., 2013; Brown, 2015; Bukhari & Rizvi, 2015; Gribble et al., 2011; Maheen & Hoban, 2017; MirMohamadalile et al., 2019). Considering the role of privacy and safe space, the literature identifies a breastfeeding tent as a space where mothers can avail themselves privacy and one-to-one breastfeeding counselling from healthcare professionals (Ayoya et al., 2013; UNICEF, 2010).

The presence or absence of breastfeeding culture, people’s attitudes toward breastfeeding, and acceptance of breastfeeding in the setting are reported to hold considerable influence on breastfeeding practices (Hausman, 2004; Schmied & Lupton, 2001). In this study, the breastfeeding culture in the village (as a capability in the external environment) was promoting maternal agency to sustain their breastfeeding practices for up to 3 years. In view of this cultural practice, displaced mothers were offered nutritious food, breastfeeding guidance, and assistance in household chores by their mothers-in-law, sisters-in-law, and neighbors which facilitated their capabilities and functioning surrounding breastfeeding. Despite the existence of gender disparity surrounding child-feeding practices in other parts of Pakistan and the South-Asian context (Fledderjohann et al., 2014; Jatrana, 2003; Miller, 1997), the finding suggested breastfeeding was viewed as the best feeding option for both genders, hence breastfeeding culture was serving as an avenue to maintain and promote gender equality. Besides strengthening displaced mothers’ capabilities in the internal environment, it was also enabling them to use their agency, independence, control, and ethical reasoning surrounding their breastfeeding decisions regardless of their child’s gender. The role of breastfeeding culture during natural disasters is well acknowledged in the literature as an avenue to promote the health and well-being of the mother-child dyad (Wolf, 2008).

In this study, various cultural myths, beliefs, and practices surrounding child feeding were, directly and indirectly, affecting maternal agency surrounding breastfeeding. In the context of Chitral where avenues to access health care support were limited, spiritual healing and practices based on cultural beliefs and practices were preferred. Family members, community people, and spiritual healers in the social network of mothers were the key providers of these practices. In this study displaced mothers who could not sustain their breastfeeding practices wished to receive spiritual support. Although the interrelationship and effects of spiritual rituals on breastfeeding practices are under-researched (Hirani & Ratushniak, 2022), spiritual practices are viewed as avenues to promote mental relaxation and socialization during stressful periods of disaster and displacement. Several studies undertaken with survivors of disaster acknowledge that spirituality, spiritual practices, and faith-based interventions facilitate building resilience, enhancing coping, and promoting the reintegration of survivors of disaster into normal life (Alawiyah et al., 2011; Jang & LaMendola, 2007; Jang & Wang, 2009; Lawson & Thomas, 2007; Ramsay et al., 2010; Subandi et al., 2014).

#### Limitations of this study

Due to unstable roads in the northern region, volatile political circumstances, and active disasters, data could not be collected from villages in the upper Chitral and other northern regions of Pakistan that are also severely affected by natural disasters. The scope of this study was to include displaced mothers who were residing in a variety of disaster relief camps at the time of fieldwork. To prevent recall bias, mothers who have returned to stable houses and those who have never lived in disaster relief camps were excluded. This exclusion criterion may have potentially affected the richness of the data. In the northern region of Pakistan, there are various underserved areas and zones where many families affected by armed conflicts are living in relief camps. In view of the political situations and safety issues, the scope of this study was limited to communities affected by natural disasters and residing in a variety of disaster relief camps.

## Conclusion and Recommendations

The critical ethnographic study uncovered a range of sociocultural factors that shape the breastfeeding practices of internally displaced mothers in disaster relief camps. Informal support, formal support, breastfeeding culture in the village, and spiritual practices facilitated the breastfeeding practices of internally displaced mothers. On the other hand, lack of privacy, cultural beliefs, and practices (food restrictions, belief in supernatural forces, and encouragement of breastmilk substitutes), covert oppression, lack of healthcare support, and family circumstances served as barriers to the breastfeeding practices of internally displaced mothers residing in disaster relief camps of Chitral, Pakistan. The findings of this study underscored that interdisciplinary and multidisciplinary efforts are essential to sustaining the breastfeeding practices of mothers residing in disaster relief camps.

The recommended system-level interventions in the practice settings include the empowerment of local people, the establishment of well-being camps to offer preventive, curative, and rehabilitation services, the creation of women/breastfeeding-friendly spaces (breastfeeding tents) during and after the disaster, and the provision of safe, accessible, and clean spaces for prayers and religious rituals. Promoting breastfeeding culture is important through various avenues (chat sessions in the neighborhood, mass media, use of flyers in the local language, announcements in prayer halls, and home visits to disaster-affected families). This system-level intervention is important in clarifying myths about breastfeeding, sharing the risks associated with the use of breastmilk substitutes (cow’s milk, tea, and formula milk), and communicating the benefits of breastmilk in general but specifically during a natural disaster. It is also recommended that spiritual/religious leaders must be involved as key stakeholders in promoting a breastfeeding culture in disaster relief camps. Religious beliefs of the displaced communities must be respected without any prejudice/ stigmatization, and breastfeeding mothers must be encouraged to seek spiritual support during stressful times of disaster and displacement.

Moreover, there must be a local, provincial, and federal policy to support the privacy needs of breastfeeding mothers affected by disaster and displacement. In view of this policy, displaced mothers who wish to sustain their breastfeeding practices must be offered a baby-friendly space during the period of disaster, displacement, and settlement in the disaster relief camps/temporary housing. Additionally, local institutions working for disaster-affected families must have a policy to offer culturally-sensitive, gender-sensitive, and context-specific services to disaster-affected families. Considering this policy, all service providers (mainly relief agency workers and health care providers) must consider the sociocultural beliefs, practices, and norms of the disaster-affected families, especially in planning and executing a variety of programs for displaced mothers who wish to sustain their breastfeeding practices. Considering the religious and cultural diversity in Chitral, at the local and provincial levels there must be a policy to facilitate disaster-affected families and communities in undertaking religious rituals and in seeking spiritual healing in view of their cultural norms, beliefs, and practices surrounding breastfeeding. Considering the existence of covert oppression and gender insensitivities during the period of disaster and displacement, awareness-raising campaigns must be initiated to promote the capabilities, functioning, and well-being of displaced mothers. Moreover, there must be a policy surrounding safeguarding the welfare, rights, and safety of displaced mothers affected by disaster and displacement.
